# A novel risk score for hepatocellular carcinoma in Asian cirrhotic patients: a multicentre prospective cohort study

**DOI:** 10.1038/s41598-018-26992-3

**Published:** 2018-06-05

**Authors:** Kung-Hao Liang, Sang Hoon Ahn, Hye Wong Lee, Ya-Hui Huang, Rong-Nan Chien, Tsung-Hui Hu, Kwang-Huei Lin, Christopher Sung-Huan Yeh, Chao-Wei Hsu, Chih-Lang Lin, Tai-Long Pan, Po-Yuan Ke, Ming-Ling Chang, Chau-Ting Yeh

**Affiliations:** 1 Liver Research Center, Chang Gung Memorial Hospital, Linkou, Taiwan; 2 Medical Research Department, Taipei Veterans General Hospital, Taipei, Taiwan; 3 Department of Internal Medicine, Institute of Gastroenterology, Yonsei University College of Medicine, Seoul, Korea; 4 Liver Research Unit, Keelung Chang Gung Memorial Hospital, Keelung, Taiwan; 5 Division of Hepatogastroenterology, Department of Internal Medicine, Kaohsiung Chang Gung Memorial Hospital, Kaohsiung, Taiwan; 6 Department of Cognitive Science, College of Letters and Science, University of California, Los Angeles, USA; 7 Molecular Medicine Research Center, Chang Gung University, Taoyuan, Taiwan

## Abstract

Liver cirrhotic patients suffer from a seemingly unpredictable risk of hepatocellular carcinoma (HCC). Here, an HCC risk score *R* (0 ≦ *R* ≦ 1) was derived from commonly tested haematological and biochemical parameters. In the score-derivation Taiwanese cohort (144 cirrhosis versus 48 HCC-remission patients), the score had an area-under-the-curve (AUC) of 0.70 (95% confidence interval [CI], 0.61–0.78, P < 0.001). When validated in a Korean cohort (78 cirrhosis versus 23 HCC-remission patients), the AUC was 0.68 (CI, 0.56–0.80, P = 0.009). In a multicentre prospective cohort (478 cirrhotic patients prospectively followed for HCC occurrence), the hazard ratio with respect to *R* was 2.344 (CI = 1.183–4.646, P = 0.015). The cumulative incidences of HCC at two years after patient enrolment were 9.6% and 1.7% for the high-risk (*R* ≧ 0.5) and low-risk (*R* < 0.5) groups, respectively (P < 0.001). At the end of the study, the incidences were 10.9% and 5.0%, respectively (P = 0.012). The majority of HCCs (23/26) in the high-risk group emerged within the first two years of follow-up. In conclusion, an HCC risk score was developed for cirrhotic patients that effectively predicted HCC in a prospective cohort study.

## Introduction

Hepatocellular carcinoma (HCC) is the sixth most common solid malignancy and the second leading cause of cancer-related death worldwide^1^. HCC oncogenesis is a complex process involving multiple aetiologies, including chronic hepatitis B (CHB)^2^, chronic hepatitis C (CHC)^3^, diabetes^4^, non-alcoholic steatohepatitis^5^, obesity, and alcoholic liver diseases^6^. Viral hepatitis is the predominant aetiology of HCC in Asia due to the high prevalence of CHB and CHC. Fortunately, the prevalence of CHB is decreasing due to the consistent execution of mass vaccination programmes in areas such as Taiwan^7^ and South Korea^8^. Additionally, potent antiviral treatments have been developed and widely used for the treatment of CHB^2^ and CHC^3^. As viral hepatitis has been gradually brought under control, non-viral factors involved in the occurrence of HCC are gaining increasing attention.

Liver cirrhosis often precedes the occurrence of HCC. Liver cirrhotic patients suffer from an increased yet seemingly unpredictable risk of hepatocellular carcinoma. A prospective study in Japan demonstrated that the three-year cumulative risk of liver cancer was 12.5% in cirrhotic patients, which is in contrast to 3.8% in non-cirrhotic hepatitis patients^9^. Thus, precautionary measures of HCC, such as regular surveillance by ultrasonography, are critical in cirrhotic patients. In U.S.^6^ and European^10^ guidelines, it is suggested that one or more screenings by ultrasonography or computer tomography should be performed per year for cirrhosis patients. A recent study in Taiwan demonstrated that mass screening by ultrasonography improved early detection of HCC and thus reduced the associated mortality^11^. However, a sonography screening programme requires intensive labour from radiologists or clinical hepatologists and is therefore economically and pragmatically unachievable. An efficient and cost-effective screening method for early or even “imminent” HCC remains an unmet medical need.

A few exploratory-stage biomarkers have been reported to discern HCC from cirrhosis. A prospective study on 114 hepatitis C virus-related cirrhosis patients revealed that insulin-like growth factor 1 (IGF-1) is inversely correlated with the onset of HCC^12^. The reduction of IGF-1 level preceded the diagnosis of HCC by 9.3 months^12^. A retrospective, exploratory comparison of HCC and cirrhotic patients revealed that fucosylated kininogen, fucosylated α-1-antitrypsin and Golgi protein 73 can all distinguish HCC from cirrhotic patients, particularly when these biomarkers are combined together^13^. Serum asialo-alpha1-acid glycoprotein concentrations were also found to be indicative of HCC among cirrhotic patients^14^. Another glycoprotein, clusterin, was shown to be elevated in HCC compared with HBV-related cirrhosis^15^. Additionally, a nomogram was developed for hepatitis C virus-related cirrhosis patients to predict HCC occurrence^16^. With the exception of the first study (IGF-1), all these studies were performed in a retrospective manner and demonstrated the ability to discern established HCC from cirrhosis. The performance of these markers in the prospective prediction of HCC occurrence in liver cirrhosis patients is unknown.

We were thus motivated to derive an HCC risk score using commonly evaluated haematological and biochemical factors and validate the score in a prospective study. This risk score did not incorporate virological factors because a great majority of cirrhosis patients are currently undergoing effective antiviral treatments, and thus, in most of these patients, the virological factors cannot be assessed. Previously established risk scores that included virological factors, such as viral loads and viral genotypes, could no longer be calculated^17^.

## Methods

### Patients

**Figure 1:**
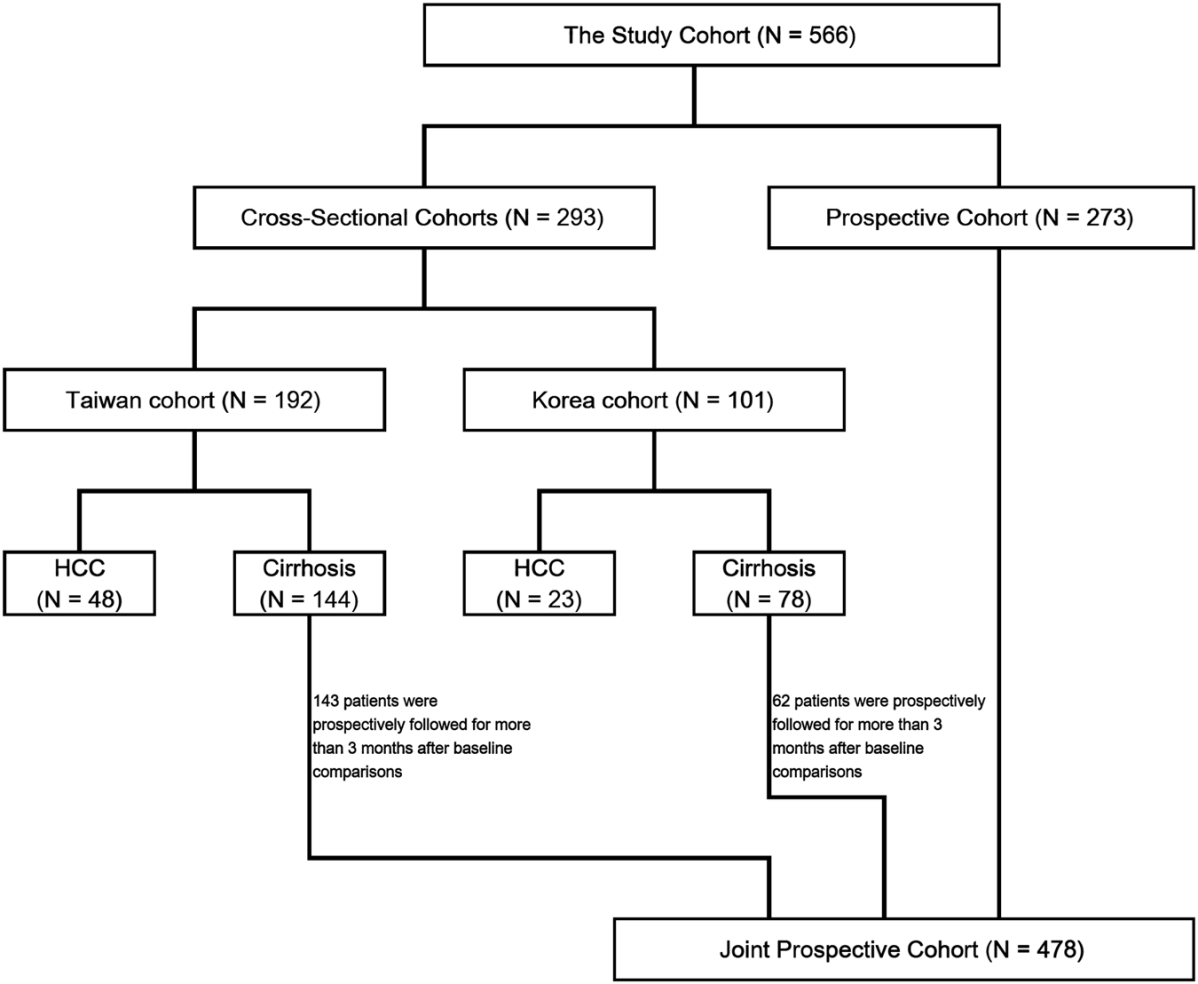
Study design and patient cohorts enrolled in this study.

This study was conducted under the approval of Institutional Review Board of Chang Gung Medical Center, Taiwan and Yonsei University College of Medicine, Korea. Taiwanese patients were recruited from three sites: Keelung, Linkou and Kaohsiung Chang Gung Memorial Hospitals (which are located in the northern, central-northern and southern parts of Taiwan, respectively). Korean patients were recruited from the Yonsei University Severacne Hospital (See Fig. 1 for deposition for all patients enrolled). Written informed consent was obtained from all patients enrolled, and the study was conducted in accordance with the Guidelines for Good Clinical Practice and the applicable laws and regulations. A two-step design was devised. The first step was a cross-sectional analysis where patients were enrolled in Taiwan (N = 192) and Korea (N = 101) for score derivation and validation, respectively (Table 1). The scores were calculated using baseline haematological and biochemical data, which were assayed at recruitment. The Taiwanese cohort comprised 144 liver cirrhosis patients who never developed HCC and 48 liver cirrhosis patients who were diagnosed as early stage HCC (Barcelona Clinic Liver Cancer [BCLC] stage A) and had achieved complete remission after surgical resections. The Korean cohort comprised 78 liver cirrhotic patients and 23 HCC patients.

**Table 1:** Age, gender and viral markers in patients recruited for this study.

	Cross-sectional Cohort				Prospective Cohort
	Taiwan Cohort		Korea Cohort		Cirrhosis
	HCC	Cirrhosis	HCC	Cirrhosis	
Subject number	48	144	23	78	273
Age	64.96 ± 9.45	58.77 ± 12.04	62.09 ± 8.62	60.24 ± 10.59	59.49 ± 10.68
Gender-Male	40 (83.33%)	98 (68.06%)	16 (69.57%)	54 (69.23%)	177 (64.84%)

The second step was a prospective study, where 143 liver cirrhosis patients of the Taiwanese cohort and 62 of the Korean cohort were subsequently followed for at least 3 months (Fig. 1). An independent, non-overlapping cohort of 273 liver cirrhotic patients was also enrolled, increasing the number of prospectively followed patients to 478 (Fig. 1). These patients were all enrolled between January, 2013 and December, 2015 and subsequently followed until March, 2017, or the occurrence of HCC, whichever came first. Patients who developed HCC within the first three month of follow-up were excluded from the analysis. Baseline measurements were performed at the time of enrolment, and the HCC risk score derived from the first step was calculated using these baseline measurements.

Cirrhosis was diagnosed by either (i) liver biopsy or (ii) ultrasonography imaging criteria^18^ plus one of the following: the presence of oesophageal varices by endoscopy, a Fibroscan stiffness value > 12 kPa, or aspartate transaminase (AST)-to-platelet ratio index (APRI) > 1. In CHB-related cirrhosis patients, life-long antiviral treatment was provided if HBV DNA level > 2000 IU/mL was detected. When included in this study, all CHB patients had HBV DNA level < 500 IU/mL. The efficacy of peg-interferon-based antiviral therapy in CHC-related cirrhotic patients was not satisfactory, and the side effects outweighed its benefit. Direct-acting antivirals were not covered by health insurance in Taiwan until 2017. Thus, the CHC-related cirrhosis patients included in this study were not treated. These patients were in a transitional period and were waiting for insurance coverage of direct-acting antivirals.

All patients were under ultrasound surveillance (liver ultrasound examination once every three months). If the ultrasound examination was positive for liver nodule(s), then a full spectrum of examinations, including dynamic computed tomography, was performed.

HCC was diagnosed by one of the following methods: (i) echo-guided liver biopsy or fine-needle aspiration cytology, (ii) alpha-fetoprotein (AFP) > 200 ng/mL, tumour > 2 cm and typical HCC characteristics in dynamic computed tomography, or (iii) typical HCC characteristics in both dynamic computed tomography and angiography. Complete remission of HCC after treatment was determined by absence of HCC in two consecutive computed tomography examinations at least 3 months apart after therapy.

### Clinical parameters included for biosignature discovery

The following 40 clinical parameters and 7 derived variables were included for establishment of an HCC risk score in cirrhotic patients (Table 2). Biochemistry variables included aspartate aminotransferase (AST), alanine transaminase (ALT), bilirubin, alpha-fetoprotein (AFP), haptoglobin, sugar before meal (sugar AC), glycohaemoglobin, free thyroxin (T4), total protein, albumin, alpha1-globulin, alpha2-globulin, beta-globulin, gamma-globulin, albumin/globulin (A/G) ratio, apolipoprotein-A1 (Apo-A1), C-reactive protein (CRP), uric acid (UA), high-density lipoprotein (HDL), low-density lipoprotein (LDL), very low-density lipoprotein (VLDL), cholesterol, triglyceride, cholesterol/HDL ratio, LDL/HDL ratio, insulin, ferritin, ceruloplasmin, iron, total iron binding capacity (TIBC), unsaturated iron binding capacity (UIBC), complement component 3 (C3), complement component 4 (C4), sugar/insulin ratio, blood urea nitrogen (BUN), creatinine and homeostasis model assessment-estimated insulin resistance (HOMA-IR). Haemogram variables included leukocyte, haemoglobin, platelet, percentage of neutrophil, percentage of lymphocyte, percentage of monocytes, lymphocyte/neutrophil ratio, log lymphocyte/neutrophil ratio, and prothrombin time (PT).

**Table 2:** Cross-sectional receiver-operating-characteristic curve analysis of 40 biochemical and hepatological variables and 7 derived variables for the classification of liver cirrhotic patients versus early-stage HCC patients under remission.

Variables	Cirrhosis	HCC	AUC	95% CI	Asymptotic P
AST (IU/L)	43.33 ± 27.60	47.69 ± 45.52	0.508	0.413, 0.603	0.865
ALT (IU/L)	39.08 ± 29.78	34.00 ± 25.76	0.429	0.331, 0.526	0.139
Bilirubin (mg/dL)	1.07 ± 0.68	1.11 ± 0.49	0.570	0.479, 0.662	0.148
AFP (ng/ml)	12.29 ± 57.53	8.88 ± 10.63	0.556	0.449, 0.663	0.242
Haptoglobin (g/dL)	65.32 ± 38.62	74.25 ± 51.84	0.530	0.428, 0.633	0.550
Sugar AC (mg/dL)	105.90 ± 35.28	114.81 ± 54.58	0.561	0.464, 0.659	0.203
Glycohemoglobin (%)	6.72 ± 7.54	6.28 ± 1.30	0.561	0.468, 0.655	0.223
Free T4 (ng/dL)	1.09 ± 0.24	1.07 ± 0.20	0.495	0.393, 0.598	0.928
Total protein (g/dL)	7.21 ± 0.82	7.25 ± 0.55	0.490	0.392, 0.588	0.835
Albumin (g/dL)	3.74 ± 2.33	3.61 ± 0.41	0.524	0.425, 0.624	0.612
Alpha1-globulin (g/dL)	0.27 ± 0.06	0.27 ± 0.06	0.464	0.363, 0.564	0.474
Alpha2-globulin (g/dL)	0.65 ± 0.12	0.67 ± 0.13	0.534	0.434, 0.634	0.501
Beta-globulin (g/dL)	0.88 ± 0.13	0.87 ± 0.16	0.482	0.374, 0.590	0.724
Gamma-globulin (g/dL)	1.92 ± 0.55	1.94 ± 0.47	0.540	0.444, 0.637	0.426
A/G ratio	0.98 ± 0.19	0.97 ± 0.18	0.466	0.365, 0.567	0.505
Apo-A1 (g/L)	1.42 ± 0.25	1.38 ± 0.27	0.437	0.337, 0.537	0.208
CRP (mg/L)	3.56 ± 2.45	3.98 ± 2.07	0.533	0.432, 0.634	0.522
Uric Acid (mg/dL)	5.95 ± 1.81	6.32 ± 1.57	0.569	0.472, 0.665	0.177
HDL (mg/dL)	51.89 ± 15.68	46.98 ± 11.07	0.404	0.313, 0.495	**0**.**047**
LDL (mg/dL)	103.05 ± 35.90	88.75 ± 31.53	0.399	0.298, 0.501	0.053
VLDL (mg/dL)	18.81 ± 10.27	23.08 ± 16.49	0.565	0.457, 0.673	0.213
Cholesterol (mg/dL)	172.27 ± 42.49	157.20 ± 39.74	0.403	0.304, 0.501	0.055
Triglyceride (mg/dL)	94.38 ± 51.36	118.00 ± 87.43	0.574	0.469, 0.679	0.146
Cholesterol/HDL ratio	3.50 ± 1.14	3.56 ± 1.14	0.522	0.423, 0.620	0.668
LDL/HDL ratio	2.93 ± 9.21	5.33 ± 15.05	0522	0.420, 0.623	0.667
Insulin (mIU/L)	10.08 ± 10.88	29.70 ± 110.80	0.610	0.517, 0.704	**0**.**022**
Ferritin (ng/mL)	236.26 ± 292.33	325.93 ± 657.51	0.531	0.428, 0.634	0.541
Ceruloplasmin (mg/dL)	24.84 ± 5.92	25.15 ± 5.62	0.503	0.400, 0.605	0.957
Iron (ug/dL)	116.71 ± 52.26	118.07 ± 51.41	0.507	0.412, 0.603	0.883
TIBC (ug/dL)	330.21 ± 56.02	315.38 ± 77.65	0.440	0.339, 0.542	0.230
UIBC (ug/dL)	209.08 ± 80.14	202.67 ± 81.43	0.465	0.364, 0.567	0.499
C3 (mg/dL)	96.33 ± 18.81	98.40 ± 21.29	0.543	0.444, 0.643	0.396
C4 (mg/dL)	18.96 ± 7.12	20.14 ± 9.57	0.508	0.405, 0.612	0.868
Sugar/Insulin Ratio	15.71 ± 10.71	13.26 ± 11.03	0.394	0.300, 0.488	**0**.**028**
Log Sugar/Insulin Ratio	1.11 ± 0.28	1.00 ± 0.35	0.394	0.300, 0.488	**0**.**028**
Blood urea nitrogen (mg/dL)	16.55 ± 10.56	16.74 ± 6.31	0.561	0.454, 0.668	0.253
Creatinine (mg/dL)	1.09 ± 1.40	0.89 ± 0.33	0.570	0.478, 0.662	0.174
HOMA-IR	2.76 ± 3.36	21.05 ± 117.48	0.604	0.511, 0.697	**0**.**031**
Leukocyte (10^9^/L)	5.39 ± 1.92	5.48 ± 1.72	0.527	0.428, 0.626	0.597
Hemoglobin (g/dL)	13.39 ± 2.08	13.82 ± 1.67	0.543	0.440, 0.646	0.400
Platelet (1000/uL)	136.32 ± 65.70	113.94 ± 52.26	0.395	0.308, 0.483	**0**.**030**
% of neutrophil	55.99 ± 11.27	60.58 ± 10.61	0.620	0.528, 0.711	**0**.**013**
% of lymphocyte	33.83 ± 10.46	30.08 ± 9.52	0.399	0.309, 0.489	**0**.**037**
% of monocytes	6.72 ± 2.21	6.14 ± 1.65	0.427	0.334, 0.520	0.154
Lymphocyte/Neutrophil ratio	0.67 ± 0.37	0.55 ± 0.30	0.393	0.302, 0.483	**0**.**026**
Log Lymphocyte/Neutrophil ratio	−0.23 ± 0.23	−0.32 ± 0.22	0.393	0.302, 0.483	**0**.**026**
Prothrombin time (s)	13.65 ± 12.47	16.09 ± 22.40	0.567	0.474, 0.661	0.195

Bold P values, P < 0.05.

### Statistical analysis

Clinical variables in different patient groups were compared using the Mann-Whitney test and area under the receiver operating characteristic curves (AUC). Confounding effects of risk scores were assessed using multivariate logistic regression. Longitudinal time-to-HCC values of different patient strata were compared using the log-rank test and visualized using the Kaplan-Meier plot. The Cox proportional hazards model was used to estimate the hazard ratios and confidence intervals of the HCC risk score. All statistical tests were two-tailed, and P-values less than 0.05 were considered statistically significant. SPSS software was used for statistical analysis (IBM, Armonk, NY).

The multivariate risk score was derived using the generalized iterative modelling (GIM) algorithm, which was published previously^19^. Briefly, GIM shares three similar components with conventional generalized linear models: (1) a **polynomial** combination of variables, denoted as t; (2) a link function connecting the values of t to the response variable *R*; and (3) a goal of optimization. The major difference between GIM and generalized linear models lies in (1), where t in a generalized linear model is defined as a **linear** combination of variables joined together by weighted additions “+” regardless of whether the variables are selected manually or by a stepwise method, such as forward or backward stepwise regressions. In contrast, t in GIM is defined as a **polynomial** combination of variables joined together by algebraic operations (i.e., addition “+” and multiplication “×”). The variables are then weighted by coefficients (b1, b2, b3….), which are rational numbers. The multiplication of variables allows the exploration of synergistic effects between variables. In this study, we used a logistic function as the link function and the U-statistics as the goal of optimization.

### Availability of data and materials

De-linked data are available to academic scientists upon request.

## Results

### HCC risk score derived from common biochemical and haematological variables

Basic demographic information about the study subjects is listed in Table 1. In the cross-sectional analysis, 48 HCC patients (under remission) and 144 liver cirrhotic patients were recruited in Taiwan (Fig. 1). This sample size was of sufficient statistical power based on an estimation shown in Supplementary Fig. 1. To develop an HCC risk score, 47 commonly tested biochemical and hepatological variables were analysed, and the AUC and P-values for asymptotic significance are listed in Table 2. Ten clinical variables reached statistical significance, including platelet (PLT), HDL, insulin, HOMA-IR, lymphocyte percentage, neutrophil percentage, sugar/insulin ratio (SIR) and lymphocyte/neutrophil ratio (LNR) in both the original and logarithmic forms. Using the GIM algorithm, a model of the HCC risk score (R) was derived from the above variables.

R was defined as a logistic function of t:

1 $${\rm{R}}={{\rm{e}}}^{{\rm{t}}}/({{\rm{e}}}^{{\rm{t}}}+1)$$

where t is a function of clinical variables:

2 $$\begin{array}{ccc}{\rm{t}} & = & -\,{\rm{0.005}}\times {\rm{PLT}}-{\rm{0.029}}\times {\rm{HDL}}-{\rm{0.376}}\times {\mathrm{log}}_{{\rm{10}}}({\rm{SIR}})+{\rm{0.854}}\times {\mathrm{log}}_{{\rm{10}}}({\rm{LNR}})\\  &  & -\,{\rm{0.015}}\times {\rm{PLT}}\times {\rm{LNR}}-{\rm{0.062}}\times {\rm{SIR}}\times {\rm{LNR}}+{\rm{4.253}}\end{array}$$

**Figure 2:**
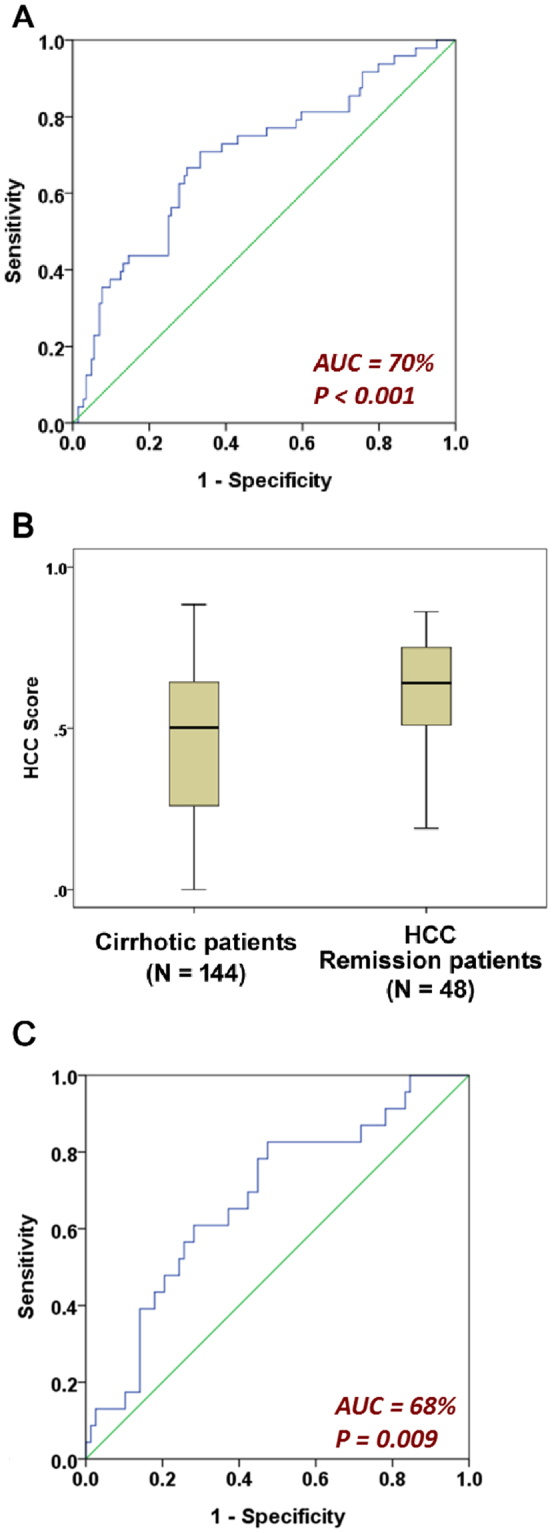
Performance of the optimized HCC Risk score *R* in classifying liver cirrhotic and HCC patients recruited in Taiwan and Korea. (**A**) The receiver operating characteristic curve of ***R*** in classifying liver cirrhotic (N = 144) and HCC (N = 48; in remission) patients recruited in Taiwan; (**B**) the distributions of ***R*** in the two patient groups. The 25^th^ percentile, median and 75^th^ percentile of the cirrhotic group were 0.25, 0.5 and 0.65, respectively. The values for the HCC group were 0.5, 0.64 and 0.75, respectively. (**C**) A validation of ***R*** in classifying liver cirrhotic (N = 78) and HCC (N = 23; in remission) patients recruited in Korea.

The value of t was determined by weighted addition of clinical variables and the multiplication of variables (such as PLT × LNR and SIR × LNR), a feature that distinguishes the GIM methodology from conventional generalized linear models. The constant term of the model was manually adjusted, making the median risk score of the 144 cirrhotic patients 0.5. The AUC was 70% (P < 0.001, Fig. 2A). The 25^th^ percentile, median and 75^th^ percentile HCC risk scores of the cirrhotic patients were 0.275, 0.5 and 0.65, respectively. In contrast, those values in the HCC group were 0.5, 0.64 and 0.75, respectively (Fig. 2B). In view of risk score distribution of the two groups, we considered liver cirrhotic patients with an HCC risk score (R) < 0.5 to carry a low risk and patients with a risk score ≧ 0.5 to carry a high risk.

After the model was constructed, we created two Apps called QuickMed that are freely downloadable online for calculating the HCC risks conveniently in iPhones and Android devices. The model has not been changed.

### Cross-sectional validation of the HCC risk score

An independent cohort of liver cirrhotic (N = 78) and HCC (N = 23) patients was recruited in Korea to validate the performance of the HCC risk score. The AUC was 68% (P = 0.009, Fig. 2C), suggesting that the HCC risk model was applicable to patients of another ethnicity in Asia.

### Prospective validation of the HCC risk score

**Figure 3:**
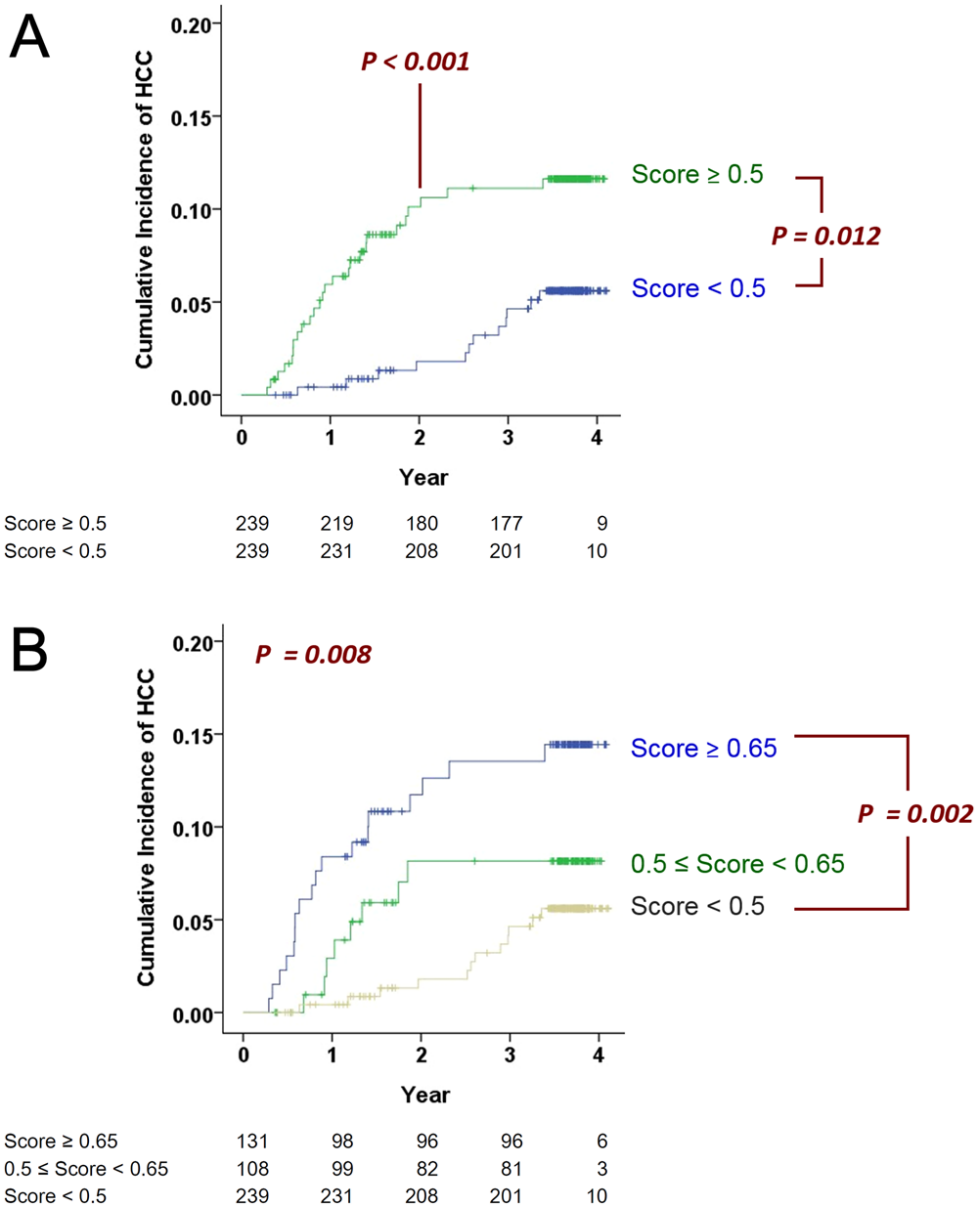
Predictive values of the HCC risk score in prospective longitudinal follow-up studies. (**A**) The Kaplan-Meier plot of 478 liver cirrhotic patients stratified by the baseline HCC risk score (Score ≥ 0.5, N = 239; Score < 0.5, N = 239). At the end of two years, P < 0.001. At the end of the study, P = 0.012. (**B**) Patients were further stratified into three risk categories: Score ≥ 0.65 (N = 131); 0.5 ≤ Score <0.65 (N = 108) and Score < 0.5 (N = 239). For the three risk categories, P = 0.008. For the highest and the lowest categories, P = 0.002.

Among liver cirrhosis patients in the cross-sectional analysis, 143 patients of the Taiwanese cohort and 62 patients of the Korean cohort were subsequently followed for at least 3 months (Fig. 1). The median follow-up time of those patients who did not develop HCC was 1385.5 [131~1499] days. The median follow-up time of those with HCC was 334 [105~1238] days. Additionally, an independent cohort of 273 cirrhotic patients was recruited exclusively for the prospective analysis. The median follow-up time of those without HCC was 1340 [378~1483] days. The median follow-up time of those with HCC was 514 [120~1224] days. Cox proportional hazards model analysis on all these prospectively followed patients (total N = 478) revealed that the hazard ratio with respect to *R* was 2.344 (CI = 1.183–4.646, P = 0.015). Patients were stratified into different risk groups according to the risk scores. At the end of the second year, 23/239 (9.6%) and 4/239 (1.7%) patients in the high-risk (*R* ≧ 0.5) and low-risk (*R* < 0.5) groups developed HCC (log-rank P < 0.001, Fig. 3A). At the end of this study, 26/239 (10.9%) and 12/239 (5.0%) patients in the high- and low-risk groups developed HCCs (log-rank P = 0.012, Fig. 3A). The majority of HCCs in the high-risk group emerged within the first two years of follow-up (23/26 [88.5%]), whereas HCC in the low-risk group occurred mostly after the second year (8/12 [66.7%]) and was associated with chronic hepatitis C (6/8 [75%]). When we further stratified the high-risk patients into two groups (*R* ≧ 0.65 and 0.5 ≤ *R* < 0.65), the three strata manifested distinct cumulative incidence (P = 0.008, Fig. 3B). Patients in the highest (R ≧ 0.65) and lowest risk (R < 0.5) groups also exhibited distinct cumulative incidence (P = 0.002). The relationship between *R* and the observed occurrence of HCC is summarized in Table 3.

**Table 3:** The prospectively observed HCC events and percentages with respect to the HCC risk scores (*R*) calculated at baseline.

HCC Risk Score	Number of Patients	Followed 2 years		Followed 4 years	
		Observed HCC Events	Percentage (%)	Observed HCC Events	Percentage (%)
**All Patients**					
R ≥ 0.65	131	15	11.45	18	13.74
0.5 ≤ R < 0.65	108	8	7.41	8	7.41
0.275 ≤ R < 0.5	122	2	1.64	7	5.74
0 ≤ R < 0.275	117	2	1.71	5	4.27
**Patients in Taiwan**					
R ≥ 0.65	112	14	12.50	17	15.18
0.5 ≤ R < 0.65	88	6	6.82	6	6.82
0.275 ≤ R < 0.5	112	2	1.79	7	6.25
0 ≤ R < 0.275	101	2	1.98	5	4.95
**Patients in South Korea**					
R ≥ 0.65	19	1	5.26	1	5.26
0.5 ≤ R < 0.65	20	2	10.00	2	10.00
0.275 ≤ R < 0.5	10	0	0.00	0	0.00
0 ≤ R < 0.275	16	0	0.00	0	0.00

**Figure 4:**
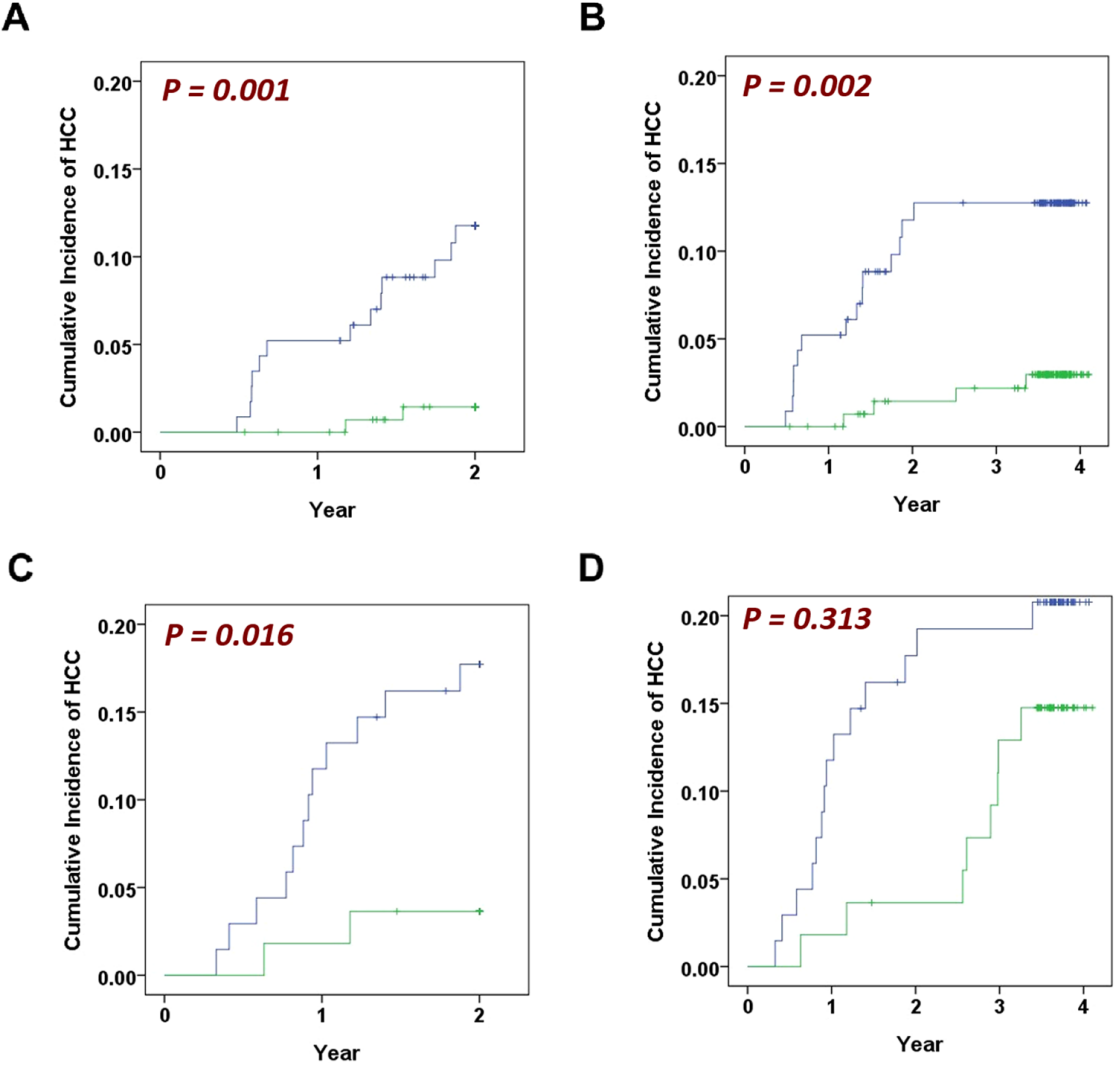
Subgroup analyses of patients with two dominant viral aetiologies. (**A**) and (**B**) Kaplan-Meier plots of HBsAg-positive patients stratified by the score *R* and followed for 2 and 4 years (P = 0.001 and = 0.002 respectively, N = 260). Blue curves: *R* ≥ 0.5, N = 115. Green curves: *R* < 0.5, N = 145. (**C**,**D**) The Kaplan-Meier plots of anti-HCV antibody-positive patients followed for 2 and 4 years (P = 0.016 and = 0.313, respectively). Blue curves: *R* ≥ 0.5, N = 68. Green curves: *R* < 0.5, N = 55.

We further analysed the subgroups of patients with the two dominant viral aetiologies. At the end of the second year, the high- and low-risk strata of patients with positive HBsAg at baseline manifested a distinct cumulative incidence (P = 0.001, Fig. 4A). Similarly, patients with positive anti-HCV antibodies exhibited different cumulative incidences (P = 0.016, Fig. 4C). At the end of the study, cumulative incidence of the high- and low-risk strata of positive-HBsAg patients remained different (P = 0.002, Fig. 4B). In contrast, the difference in patients with positive anti-HCV antibodies was lost (P = 0.313, Fig. 4D).

### Evaluation of the age and gender factors for risk assessment

Age and gender are two well-known risk factors of HCC despite the fact that their associated risk could not be mitigated by medical interventions. In fact, these two factors plus AST could already establish a risk prediction model^19^. In the REVEAL-HBV study, age and gender were incorporated into an HCC risk model established for non-cirrhotic, hepatitis B-infected patients^17^. Here, we examined whether the predictive value was improved when these two factors were incorporated into our HCC risk score *R* for cirrhosis patients. First, we employed the same risk score assignment as that used in the REVEAL-HBV study and produced the age-gender score. This score was increased by 1 for every 5-year increment of age starting from the minimum age of the cohort (31 years old), increased by 2 if the patient was male, and remained unchanged if the patient was female. The age-gender score thus retained the relative weighting of the two factors to be consistent with previous studies. Second, a multivariate logistic regression analysis was performed to evaluate the confounding effects of *R* or age-gender score in the cross-sectional analysis (Taiwanese and Korean cohorts combined, N = 293). Both scores were independently associated with HCC (*R:* adjusted P < 0.001, age-gender score: adjusted P = 0.001, Supplementary Table 1). A combined risk score was thus established by use of the multivariate logistic equation incorporating both *R* and the age-gender score as follows:

$$\begin{array}{lll}{\rm{Combined}}\,{\rm{Risk}}\,{\rm{Score}} & = & {{\rm{e}}}^{{\rm{u}}}/({{\rm{e}}}^{{\rm{u}}}+1);{\rm{u}}=2.601617\times {\rm{R}}+0.213362\\  &  & \times \,(\mathrm{age}\,-\,\mathrm{gender}\,{\rm{score}})-\mathrm{4.378152.}\end{array}$$

The AUC of classification for *R* alone was 69% (Supplementary Fig. 2A), whereas the AUC for the age-gender score was 65% (Supplementary Fig. 2B). The AUC by the combined risk score was slightly improved to 73% (Supplementary Fig. 2C). Nevertheless, this improvement did not achieve statistical significance (P = 0.452 and 0.137, respectively). The high- and low-risk strata determined by *R* manifested significantly different cumulative incidence of HCC at the end of the second year (P < 0.001, Supplementary Fig. 2D). No significant differences were identified when patients were stratified by the age-gender score alone (P = 0.065, Supplementary Fig. 2E). A significant difference was observed when the combined score was used (P = 0.003, Supplementary Fig. 2F), but the difference was smaller than that when *R* was used alone (Supplementary Fig. 2D and F). Based on the above observations, the HCC risk score *R* without the incorporation of age and gender was recommended.

## Discussion

In the present study, we discovered that the HCC risk of a liver cirrhotic patient could be calculated by commonly tested hepatological and biochemical factors, which were shown to be predictive of subsequent HCC events in a prospective study. This score could be easily evaluated in an ordinary medical facility. This score was derived with a purpose of improving the current sonography-alone screening strategy where the compliance was not satisfactory. With such a score in place, intensive sonography screening can be applied exclusively to the high-risk patients, whereas haematological and biochemical assays can be performed on the lower risk patients on a regular basis until the score indicates a high-risk stage.

Observations in the prospective study supported the proposed screening strategy incorporating the HCC score derived from haematological and biochemical factors. Most HCCs in high-risk patients emerged within the first two years of follow-up, whereas most HCCs in low-risk patients emerged after the second year of follow-up. At the end of the second year, the accumulated incidences of HCCs between the two groups exhibited the greatest difference (Fig. 3A). The 6 components of this risk score may change with time; thus, the score itself could also be altered with time. In low-risk patients, the score could increase in some patients after two years of follow-up. IN contrast, among high-risk patients, those who did not develop HCC in the first two years might exhibit a decreasing risk score thereafter. If R < 0.5, the patient is allowed to repeat this test 1–2 years later, carrying a yearly HCC risk of <1%. However, if R ≧ 0.5, the patients should be urged to accept intensive ultrasound surveillance given that the HCC risk is estimated as >5%/year.

Interestingly, when we performed the subgroup analysis of patients with two dominant viral aetiologies, we found that *R* was predictive in chronic hepatitis B patients at the 2-year and 4-year follow-up (P = 0.001 and 0.002, respectively, Fig. 4A,B). In contrast, *R* was predictive in chronic hepatitis C patients only at the 2-year follow-up (P = 0.016, Fig. 4C) but not at the 4-year follow-up (P = 0.313, Fig. 4D). The reasons for this phenomenon were unknown at this time. Currently, antiviral therapy can either suppress viral replication, such as that noted in CHB infection, or it can completely eradicate viral hepatitis, such as that noted in CHC infection. Viral suppression in CHB could impede the occurrence of HCC as demonstrated by a randomized, placebo-controlled clinical trial of lamivudine for CHB^20^.

A recent retrospective study also revealed a significant reduction in HCC risk following the availability of antiviral therapy^21^. Such a HCC-preventive effect by antiviral treatment however remained debatable in cirrhotic patients. The beneficial effect of lamivudine could be compromised with the development of drug resistance^20^. In HBeAg-negative cirrhotic patients, nucleos(t)ide analogue treatment did not reduce HCC risk^22^. Furthermore, a review combining 21 studies demonstrated that if the nucleos(t)ide analogue treated patients failed to remain in virological remission, an increased risk of HCC compared with untreated patients was identified^23^. On the other hand, interferon-based antiviral or direct-acting antiviral therapy could eradicate CHC infection and thus significantly reduce HCC risk. However, the therapeutic efficacy for interferon-based treatment was greatly reduced in patients with liver cirrhosis, and recent studies demonstrated that direct-acting antiviral therapy could increase HCC risk in cirrhosis patients^24,25^. Thus, HCC continued to develop in cirrhosis patients despite rapid advances in antiviral therapy, and HCC surveillance in cirrhosis patients remained mandatory in clinical practice.

Of the six components of this score, insulin/sugar ratio is related to diabetes, HDL is related to lipid metabolism, platelet count is associated with severity of fibrosis, and lymphocyte/neutrophil ratio is associated with clinical outcomes in many cancers, including HCC. These components are all known risk or prognosis factors in oncogenesis of HCC; thus, our results were consistent with these studies.

AFP is generally considered a diagnosis biomarker. However, some studies demonstrated that increased AFP could predict HCC occurrence. However, in our initial univariate analysis for score development (Table 2), AFP was not a significant predictor. Our HCC-remission group included patients with early HCC who achieved complete tumour eradication, which was demonstrated by computed tomography. Using this approach, AFP exhibited no significant difference between the HCC remission and cirrhotic groups. It is unclear why some studies revealed that higher AFP levels could be a predictor for HCC. It remains possible that patients with increased AFP could have already developed micro-HCC lesions that were too small to be detected. Such patients should still be considered carrying a high risk for HCC development.

This study was limited by a relatively small sample size compared with many retrospective studies^19^. Despite the collaboration of multiple medical centres in Taiwan and South Korea, recruitment of liver cirrhotic patients for a longitudinal follow-up was still difficult. The number of patients for this prospective study was less than 500. Therefore, we could only stratify the risk into 2–4 levels. The estimation of subtle risk difference with increased resolution may require a larger sample size. However, the main purpose of establishing this score is to distinguish between patients who have a high risk of imminent HCC and those harboring a low risk. The high-risk patients would be urged to receive more frequent ultrasound examination, while those with low-risks can receive the same hematological and biochemical examinations routinely (if ultrasound surveillance is difficult to implement), until the score indicates a high risk. Hence, this score is not for a final diagnosis of HCC and an AUROC of ~70% is acceptable. It may be difficult to further improve the AUROC based on current knowledge. A major reason is that some of the cirrhosis patients (no HCC developed during this study) could still develop HCC in the future follow-ups.

In conclusion, we have developed an HCC risk score exclusively composed of commonly tested clinical parameters. The risk score effectively predicted HCC occurrence in cirrhosis patients in a longitudinal prospective follow-up study combining Taiwanese and Korean patients.

## Electronic supplementary material

Supplement 1
