# Supplementary material for: A novel risk score for hepatocellular carcinoma in Asian cirrhotic patients: a multicentre prospective cohort study
Source: Sci Rep. 2018 Jun 5;8:8608. doi: 10.1038/s41598-018-26992-3 (PMC5988718; doi:10.1038/s41598-018-26992-3)
Supplement: Supplementary file 1 — Supplement 1 [file 41598_2018_26992_MOESM1_ESM.pdf]

Supplementary Information

A novel risk score for hepatocellular carcinoma in Asian cirrhotic patients: a multicentre prospective cohort study

Kung-Hao Liang,[1,2] Sang Hoon Ahn,[3] Hye Wong Lee,[3] Ya-Hui Huang,[1] Rong-Nan Chien,[4] Tsung-Hui Hu,[5] Kwang-Huei Lin,[1] Christopher Sung-Huan Yeh,[6] Chao-Wei Hsu,[1] Chih-Lang Lin,[4] Tai-Long Pan,[1] Po-Yuan Ke,[1] Ming-Ling Chang,[1] Chau-Ting Yeh[1,7*]

Supplementary Table 1. Multivariate logistic regression analysis of the HCC Risk score *R* and the age-gender score for the classification of liver cirrhotic and HCC-remission patients

	coefficient	Odds Ratio (95% CI)	P
R	2.602	13.486 (3.647 - 49.864)	<0.001
Age-Gender Score	0.213	1.238 (1.086 - 1.410)	0.001

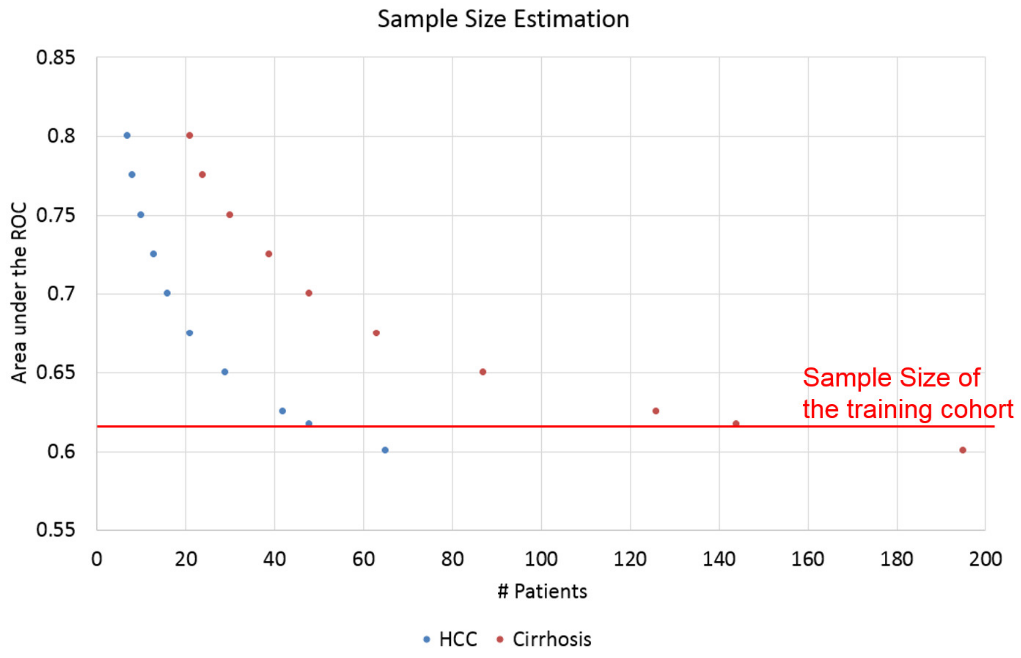

Supplementary Fig 1. The relationship between the number of patients required and the area under the receiver operating characteristic (ROC) curve. The type 1 error was 0.05 and the power was 0.8. Assuming the ratio between cirrhotic and HCC patients were 3:1. Area under the ROC ≧ 0.617 should be confidently estimated when the numbers of cirrhotic and HCC patients were 144 and 48 respectively. The sample size estimation was performed by the easyROC software (version 1.3) at http://www.biosoft.hacettepe.edu.tr/easyROC/.

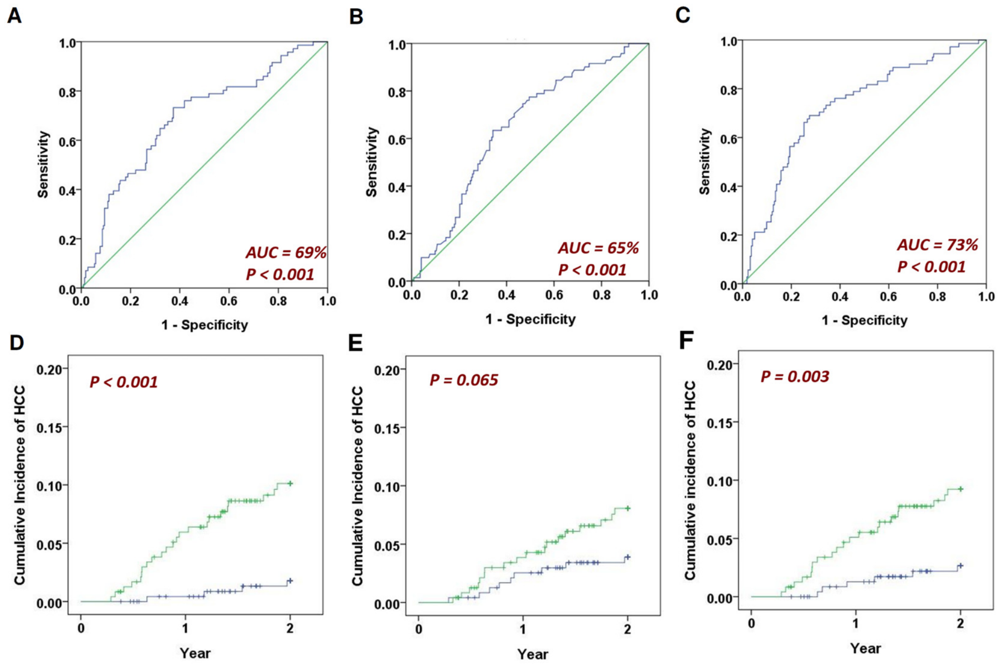

Supplementary Fig 2. Performance of patient classification and longitudinal HCC prediction. (A) – (C): The ROC of patient classification. (D)-(F):The Kaplan-Meier plots of longitudinal HCC prediction. The higher-risk patient stratum was shown in green (N = 239); the lower-risk patient stratum shown in blue (N = 239). (A) and (D): the HCC risk score *R*; (B) and (E): the age-gender score; (C) and (F): the combined score incorporating age, gender and *R*.
